# Coping Strategies in Patients with Bipolar Disorder (BD) and Major Depressive Disorder (MDD) and Their Correlation with Clusters of Psychiatric Symptoms

**DOI:** 10.3390/healthcare13091050

**Published:** 2025-05-02

**Authors:** Bianca-Oana Bucatoş, Laura Gaita, Ana-Maria Romoşan, Ion Papava, Miruna Popovici, Radu-Ştefan Romoşan, Mariana Bondrescu, Ana-Maria Cristina Daescu, Liana Dehelean

**Affiliations:** 1 Department of Neurosciences-Psychiatry, “Victor Babes” University of Medicine and Pharmacy, 300041 Timisoara, Romania; 2 Ph.D. School Department, “Victor Babes” University of Medicine and Pharmacy, 300041 Timisoara, Romania; 3 Second Department of Internal Medicine, “Victor Babes” University of Medicine and Pharmacy, 300041 Timisoara, Romania; 4 “Pius Brînzeu” Emergency County Hospital, 300723 Timisoara, Romania; 5 Clinic of Internal Medicine and Ambulatory Care, Prevention and Cardiovascular Recovery, Department VI—Cardiology, “Victor Babes” University of Medicine and Pharmacy, 300041 Timisoara, Romania; 6 Centre for Cognitive Research in Neuropsychiatric Pathology, “Victor Babes” University of Medicine and Pharmacy, 300041 Timisoara, Romania; 7 Center for Studies in Preventive Medicine, “Victor Babes” University of Medicine and Pharmacy, 300041 Timisoara, Romania

**Keywords:** bipolar disorder, major depressive disorder, coping strategy, COPE, SCL-90

## Abstract

**Background:** Adjustment to stress requires the involvement of coping strategies. Using maladaptive coping strategies may precipitate the onset or recurrence of psychiatric disorders. On the other hand, the illness itself may alter the coping mechanisms of an individual. This study aims to identify the coping strategies in patients with bipolar disorder (BD) and major depressive disorder (MDD) and determine the correlation between coping strategies and clusters of psychiatric symptoms. **Material and Methods:** Socio-demographic and clinical data were analyzed for 30 inpatients with BD and 30 inpatients with MDD. The SCL-90 questionnaire and COPE inventory were filled in by the participants. **Results:** Compared to the general population, the patients with BD had lower scores for functional coping strategies and higher scores for one dysfunctional coping strategy. The patients with MDD had lower scores for all active functional and two passive functional coping strategies. By contrast, they presented higher scores on one passive functional and one dysfunctional coping strategy. Positive reinterpretation and growth were negatively correlated with somatization, depression, anxiety, interpersonal sensitivity, hostility, and psychoticism. Behavioral disengagement was positively correlated with depression, anxiety, somatization, interpersonal sensitivity, and psychoticism. Substance use was positively correlated with the number of episodes. Distinct coping mechanisms were associated with certain symptom clusters. **Conclusions:** Although dysfunctional coping strategies may predispose to psychiatric disorders, in our study, they appear to be state-dependent rather than trait-dependent.

## 1. Introduction

Studies have shown depression as a common, heterogeneous, and important health problem across the world, with higher prevalence in women, but also increasing in men. It seems that combined self-reports and diagnostic interviews are more effective ways to assess depression [1]. The etiology of major depressive disorder (MDD) and bipolar disorder (BD) is still incompletely known. The factors involved are genetic, psychological, and familial in nature, along with negative events throughout life [2,3]. Individuals with MDD experience workplace absenteeism, suicide attempts, treatment resistance, and other comorbid conditions [3].

Children who have experienced adversities are more likely to engage in hazardous lifestyle practices, like substance use, which may lead to social and chronic health problems, making them vulnerable to depression [4]. Using alcohol as a coping mechanism for depressive symptoms in adolescence and early adulthood may result in continuing with this strategy long term if depressive symptoms persist [5].

Molecular genetics has shown that while BD is a heterogeneous illness with the core clinical features of cyclic mood and activity elevation, there are also common genetic risk factors for schizophrenia and MDD [6]. Patients with BD encounter significant life adversity and tend to have a history of early life physical or sexual maltreatment, putting them at a risk of suicide attempts and ideation [7,8]. Childhood mistreatment represents a major risk factor for developing BD in later life. Data shows that less genetic risk is needed to develop BD when childhood maltreatment factors are involved [9]. Also, results from two major cohort studies have shown that BD is the psychiatric disorder with the highest rates of completed suicide attempts [10].

Cognitive behavioral therapy (CBT) intervention provides coping strategies and techniques for addressing and correcting cognitive distortions [11]. As coping strategies are key components of psychotherapeutic interventions, particularly cognitive behavioral therapy, which targets maladaptive coping and cognitive distortions, understanding their patterns in mood disorders may inform the development of more tailored, effective non-pharmacological treatments. One major classification for coping strategies separates them into three categories: (i) coping strategies focused on the problem, such as active coping, planning, and seeking instrumental support, (ii) coping mechanisms focused on emotions, such as positive reevaluation, acceptance, a sense of humor, a return to religion, and seeking emotional support, and (iii) dysfunctional coping strategies like activities, denial, discharge, substance use, cessation of operations, and blaming oneself [12]. Another classification uses searching for social support and/or avoidance as the third category, along with emotion-focused and problem-focused (i.e., problem-solving) clusters, and is linked to the basic responses to stress [13]. It is still debatable whether these coping strategies are originally situational and/or intrinsic [13,14]. Since severe mental illnesses are extremely burdensome, they require patients to resort to coping strategies. Studies show that even under the best circumstances, many patients have recurrences or partial relapses, which result in a decrease in their functionality [15]. Thus, psychological factors, such as active, passive, and dysfunctional coping strategies, should be analyzed in patients with mood disorders [16].

The data from the literature show that both patients with BD and MDD use maladaptive coping mechanisms in stressful situations [7,17]. The identification of a cluster of coping strategies in patients can lead to an individualized non-pharmacological treatment, with the intention of designing psychoeducational and psychotherapy programs [18,19,20] that stimulate the use of adaptive coping strategies and discourage the dysfunctional ones [21,22]. The use of active coping strategies negatively correlates with the intensity of depression [23,24]. Consequently, patients with severe depression use passive and avoidant coping strategies more often [25]. In addition, in the case of patients with depressive disorders, quality of life has been critically influenced, among other factors, by the use of positive coping strategies [26]. De Beradis and collaborators suggested that in all patients with MDD, the religious coping strategy should be assessed because positive religious coping may be protective against suicidal ideation [27]. Due to some overlapping symptoms, such as impulsivity, irritability, distractibility, flight of thoughts, and affective instability, the differential diagnosis between BD and borderline personality disorder (BPD) may be difficult. BD and BPD may be considered distinct disorders that may sometimes co-occur, or they may be part of a broader spectrum [27,28,29]. Some coping strategies may be altered by disease, becoming dysfunctional [30]. In patients with BD, the presence of dysfunctional attitudes has been associated with worse personal and clinical recovery [31].

### Objectives

The present study aims to identify the most frequent coping strategies in patients with mood disorders (BD and MDD) and the possible correlations between coping strategies and psychiatric symptom dimensions, as assessed by patients. We aim to examine how specific coping strategies are associated with distinct symptom clusters, such as depression, anxiety, and somatization, as measured by the SCL-90-R. This will help determine whether these coping patterns are consistent personality traits predisposing to a certain cluster of symptoms (trait-dependent) or are influenced by the current clinical state (state-dependent) in patients with mood disorders. By linking specific coping styles to symptom dimensions (e.g., depression, anxiety, and somatization), we aim to identify clinically relevant patterns that may guide the development of targeted psychotherapeutic interventions. This approach helps differentiate whether certain strategies (e.g., avoidance or religious coping) are associated with higher symptom burdens or whether they function as maladaptive responses to distress. These findings can inform targeted therapeutic approaches, such as reinforcing certain coping strategies in patients with specific symptom profiles.

## 2. Materials and Methods

We conducted a prospective study on patients admitted to the “Eduard Pamfil” Clinic of Psychiatry, Timisoara, between the 1st and 31st of March 2021. We aimed to identify the common coping strategies in patients with BD and MDD and their correlation with clusters of psychiatric symptoms. The patients were diagnosed with BD or MDD using DSM-5 diagnostic criteria. All 60 participants were separated into two equal groups, according to their diagnosis. The control group included 33 healthy volunteers having no psychiatric disorders, closely matched by age, gender, and residence area to the clinical groups. The group included 8 males and 25 females, with a mean age of 48 years.

The inclusion criteria were: (1) inpatients with MDD or BD (currently in depressive episodes), (2) aged between 18 and 75 years, (3) the ability to understand the content of the interview and to assess the scales, and (4) the ability to communicate with the interviewers and to provide written informed consent. The exclusion criteria were: (1) patients with current substance-induced or organic mood disorders, (2) patients with cognitive impairment, (3) patients with developmental delay, (4) patients with incomplete or incorrectly filled assessment scales, (5) patients who were illiterate, and (6) patients who refused to sign the informed consent form.

The socio-demographic and clinical data were analyzed for 30 inpatients with BD and 30 inpatients with MDD. The following data were analyzed:

(i) Socio-demographic data: age, gender, and area of residence.
(ii) Clinical data: age at disorder onset, number of episodes, the average length of hospitalization, psychiatric symptoms assessed by the patients using the SCL-90-R, and preferred coping strategies, using the COPE inventory.

The Symptoms Checklist Revised (SCL-90-R) is an auto-evaluation, multidimensional inventory of symptoms developed by Derogatis after the Hopkins Symptom Checklist (HSCL) [32]. It consists of 90 items, each assessed on a 5-point suffering scale from none at all (0) to extreme (4). Through this scale, nine main dimensions of symptoms, plus three global pathology indices, are assessed. The main symptomatic groups include somatization, obsession–compulsion, interpersonal sensitivity, depression, anxiety, hostility, anxiety–phobia, paranoid ideation, and psychoticism. The SCL-90-R was proved to be a reliable scale for evaluating psychiatric symptoms in the Romanian population, with high internal consistency, expressed by a Cronbach alpha index above 0.95 [33].

The COPE Inventory is a 60-item questionnaire assessing 15 coping mechanisms by answering questions concerning a stressful situation. The coping strategies may be active (active coping, planning, suppression of competing activities, restraint, use of instrumental social support, and humor) or passive (use of emotional social support, positive reinterpretation and growth, acceptance, religious coping, and denial). Four of them are classified as dysfunctional: mental disengagement, behavioral disengagement, focus on and venting of emotions, and substance use. The 15 types of coping strategies are divided into four factors. The first is defined as a mechanism for adapting to focus on issues, including planning, active coping, and suppression of competing activities. The second factor is defined as an approach focused on emotion and includes positive reinterpretation and growth, restraint, acceptance, and a religious approach. The third factor is defined as coping with social support and includes the use of instrumental social support and a focus on and venting of emotions. The fourth factor, defined as avoidance coping, includes mental or behavioral disengagement and denial. To assess the patients’ coping strategies, they are instructed to answer questions concerning a stressful, problematic situation. In the case of evaluating situational coping, the patients will respond by referring to a specific event. A meta-analysis of correlations between coping and adaptation strategies for COPE results showed that 8 out of 17 subscales were significantly correlated with suffering [34], such as depressive symptoms, anxiety, negative feelings, stress, and physical symptoms. Eleven out of fifteen subscales were significantly correlated with well-being. In particular, denial and behavioral and mental disengagement have been associated with high levels of stress and low levels of well-being, and self-blame has been associated with increased levels of suffering. Thus, these strategies can be useful for predicting negative states, such as stress and suffering, but also well-being. The questionnaire was validated for use in the Romanian population, having good internal consistency expressed by an average Cronbach’s alpha coefficient of 0.74 [35].

### Statistical Analysis

The statistical analysis was performed using IBM SPSS Statistics for Windows, version 20. The independent-sample *t*-test was used to compare the results of the scales applied to the two patient groups. Using the one-sample *t*-test, the results obtained by the patients were compared with those recorded by the healthy control group. The control group was enrolled to fill out the SCL-90-R questionnaire and COPE inventory, after having their written informed consent. The Pearson’s correlation coefficient (r) was used to test the correlations/associations between the numerical variables. The differences between category variables were investigated using the chi-square test. The statistical significance threshold was set at *p* < 0.05.

## 3. Results

### 3.1. Socio-Demographic and Clinical Characteristics

A total of 60 patients were enrolled in the present study. The socio-demographic and clinical characteristics of the two samples are presented in Table 1.

### 3.2. Comparisons Between Study Groups

#### 3.2.1. SCL-90: BD vs. MDD

The patients with BD had significantly lower scores than those with MDD for the following SCL-90 items: interpersonal sensitivity (t(54) = −2.37, *p* = 0.02, 95% CI: −0.87; −0.07) and depression (t(58) = −3.36, *p* = 0.001, 95% CI: −1.23; −0.31), as is presented in Table 2.

#### 3.2.2. COPE: BD vs. Control Group

The patients with BD had significantly lower scores than the general population for the following coping strategies: positive reinterpretation and growth (t(29) = −2.40, *p* = 0.02, 95% CI: −2.42; −0.19), mental disengagement (t(29) = −2.48, *p* = 0.01, 95% CI: −1.65; −0.16), and active coping (t(29) = −2.18, *p* = 0.03, 95% CI: −2.23; −0.07). They presented higher scores than the general population regarding coping strategy related to the focus on and venting of emotions: t(29) = 3.06, *p* = 0.005, 95% CI: 0.59; 2.99.

#### 3.2.3. COPE: MDD vs. Control Group

The patients with MDD had significantly lower scores than the healthy control group for the following coping strategies: positive reinterpretation and growth (t(29) = −6.74, *p* < 0.0001, 95% CI: −3.92; −2.09), mental disengagement (t(29) = −4.27, *p* < 0.0001, 95% CI: −2.23; −0.78), use of instrumental social support (t(29) = 3.73, *p* = 0.001, 95% CI: −2.88; −0.83), active coping (t(29) = −8.75, *p* < 0.0001, 95% CI: −3.60; −2.23), denial (t(29) = −6.43, *p* < 0.0001, 95% CI: −2.83; −1.46), humor (t(29) = −4.53, *p* < 0.0001, 95% CI: −3.80; −1.43), restraint (t(29) = −2.23, *p* = 0.03, 95% CI: −2.33; −0.10), suppression of competing activities (t(29) = −7.66, *p* < 0.0001, 95% CI: −2.63; −1.52), and planning (t(29) = −3.86, *p* = 0.001, 95% CI: −3.77; −1.16). They had higher scores than the general population for the following coping strategies: religious coping (t(29) = 5.64, *p* < 0.0001, 95% CI: 1.65; 3.53) and behavioral disengagement (t(29) = 4.12, *p* < 0.0001, 95% CI: 0.93; 2.78).

#### 3.2.4. COPE: BD vs. MDD

The patients with BD had significantly higher scores than those with MDD for the following coping strategies: positive reinterpretation and growth (t(58) = 2.41, *p* = 0.02, 95% CI: 0.29; 3.10); use of instrumental social support (t(58) = 2.22, *p* = 0.03, 95% CI: 0.16; 3.03); active coping (t(49) = 2.87, *p* = 0.007, 95% CI: 0.51; 3.02); denial (t(46) = 3.10, *p* = 0.003, 95% CI: 0.74; 3.51); humor (t(55) = 3.07, *p* = 0.003, 95% CI: 1.01; 4.79). The patients with MDD had significantly lower scores than those with BD for the following coping strategies: religious coping (t(48) = −2.48, *p* = 0.02, 95% CI: −3.91; −0.41) and behavioral disengagement (t(58) = −3.23, *p* = 0.002, 95% CI: −3.12; −0.73). (Table 3)

### 3.3. Correlations Between SCL-90 and COPE

Next, we performed correlations between the SCL-90’s nine symptom dimensions and the coping strategies (Table 4).

The SCL-90 somatization score correlated negatively with the following coping strategies: positive reinterpretation and growth (r = −0.35, *p* = 0.005), humor (r = −0.27, *p* = 0.03), and planning (r = −0.28, *p* = 0.02). Thus, the higher the somatization scores, the fewer patients engaged in the use of these coping strategies. On the other hand, high somatization scores correlated positively with the following coping strategies: focus on and venting of emotions (r = 0.28, *p* = 0.02), religious coping (r = 0.27, *p* = 0.03), and behavioral disengagement (r = 0.37, *p* = 0.003).

The obsession–compulsion score on the SCL-90 scale correlated negatively with the following stress coping strategies: positive reinterpretation and growth (r = −0.36, *p* = 0.04), focus on and venting of emotions (r = −0.35, *p* = 0.007), and humor (r = −0.27, *p* = 0.03). Thus, the higher the scores for obsession–compulsion, the less patients used the coping strategies mentioned above. The scores obtained by patients with obsessive–compulsive symptoms (SCL-90) were positively correlated with behavioral disengagement (COPE): r = 0.39, *p* = 0.002.

The interpersonal sensitivity score on the SCL-90 scale was negatively correlated with the following coping strategies: positive reinterpretation and growth (r = −0.45, *p* < 0.0001), active coping (r = −0.26, *p* = 0.04), denial (r = −0.28, *p* = 0.03), and humor (r = −0.26, *p* = 0.03). Interpersonal sensitivity correlated positively with behavioral disengagement (r = 0.39, *p* = 0.002).

The SCL-90 depression score correlated negatively with the following stress coping strategies: positive reinterpretation and growth (r = − 0.34, *p* = 0.007) and planning (r = −0.28, *p* = 0.02). Depression correlated positively with behavioral disengagement (r = 0.49, *p* < 0.0001).

The anxiety score on the SCL-90 was negatively correlated with the following stress coping strategies: positive reinterpretation and growth (r = −0.41, *p* = 0.001) and humor (r = −0.27, *p* = 0.03). Anxiety correlated positively with scores on religious coping (r = 0.25, *p* = 0.04) and behavioral disengagement (r = 0.48, *p* < 0.0001).

The hostility score correlated negatively with the following stress coping strategies: positive reinterpretation and growth (r = −0.47, *p* < 0.0001), humor (r = −0.29, *p* = 0.02), and planning (r = −0.25, *p* = 0.04).

The SCL-90 phobic anxiety score was negatively correlated with the score for positive reinterpretation and growth (r = −0.35, *p* = 0.005). Phobic anxiety was positively correlated with mental disengagement (r = 0.36, *p* = 0.004).

The score for paranoid ideation correlated positively with the focus on and venting of emotions (r = 0.27, *p* = 0.03).

The SCL-90 psychoticism score correlated negatively with the following coping strategies: positive reinterpretation and growth (r = −0.39, *p* = 0.002) and active coping (r = −0.27, *p* = 0.03). Psychoticism correlated positively with behavioral disengagement (r = 0.37, *p* = 0.003).

A positive correlation was found between the number of episodes (recurrences) and the coping strategy of substance use: r = 0.37, *p* = 0.003. Thus, the higher the number of recurrences, the more frequently patients adopted substance use as a coping strategy in stressful situations.

No significant differences were found between men and women in the COPE and SCL-90 scores, as well as between patients living in urban and rural areas in terms of scores on the psychometric assessment scales. There were also no significant correlations (*p* > 0.05) found between age at onset, number of episodes, the average length of hospitalization, and scores on the COPE or SCL-90.

## 4. Discussion

The patients with MDD scored significantly lower for all active functional coping mechanisms, as well as for passive functional coping strategies, such as denial, positive reinterpretation, and growth, when compared to the general population. Contrarily, these patients had higher scores than the general population for religious coping (passive functional coping strategy) and behavioral disengagement (dysfunctional coping strategy). Our results are supported by another study conducted on patients with MDD [35]. In our study, patients with depression had neither higher nor lower scores regarding the use of emotional social support. Using the HSCL to distinguish between depressive and non-depressive patients, Coyne and collaborators found that the former used seeking emotional and informational support as a coping strategy [36].

Religious coping and behavioral disengagement are more frequently used by patients with MDD compared to the general population. Compared to the patients with MDD, the patients with BD presented significantly higher scores for the following active functional coping strategies: use of instrumental social support, active coping, and humor, respectively, and passive functional coping strategies like denial, positive reinterpretation, and growth. The patients with BD had significantly lower scores on the SCL-90-R for interpersonal sensitivity than patients with MDD. A study showed that when comparing patients with euthymic unipolar and bipolar disorders, the latter had higher scores for active coping, instrumental social support, planning, and positive reinterpretation [37]. Moreover, patients with BD used more task-oriented and avoidant coping strategies than patients with depressive disorder, although the difference became less significant when the intensity of depressive symptoms was higher [38].

The patients included in our study used passive functional coping strategies (religious coping) and dysfunctional coping strategies (focus on and venting of emotions and behavioral disengagement) more frequently than active functional coping mechanisms (positive reinterpretation and growth, humor, and planning). Compared to the general population, the patients with BD had lower scores when it came to functional active (such as active coping) and passive (such as positive reinterpretation and growth) coping strategies. At the same time, the patients with BD had significantly higher scores than the general population for dysfunctional coping strategies, like the focus on and venting of emotions. A study found that patients with bipolar disorder in manic episodes used predominantly active coping when compared to depressed, remitted, or healthy patients [39].

Regarding the correlations between the SCL-90-R symptomatic dimensions and the COPE adjustment strategies, the connections may be understood in two possible ways. Either a cluster of coping strategies predisposes patients to one or more symptomatic dimensions, or the disorder itself (BD or MDD) alters the patients’ coping strategies. Several studies emphasize the time-stability of coping strategies, differentiating between dimensional (trait-dependent) and dispositional (state-dependent) coping mechanisms [40,41].

In our study, the scores for several SCL-90-R symptomatic clusters showed positive and negative correlations with specific coping strategies. To understand the nature (trait- or state-dependent) of these correlations, we looked for differences between the scores of patients with BD and MDD. If a coping strategy is more frequently used by patients diagnosed with either BD or MDD, the coping strategy may be the result of the patient’s personality (trait), type of mood disorder, or the type of episode (clinical state). Then, for each identified coping strategy, we compared the patients’ scores (either BD or MDD) with the scores found in the general population. If a certain coping strategy was more frequent in the patients’ sample than in the general population, it is more probable to be state-dependent rather than trait-dependent.

Somatization scores on the SCL-90-R were negatively correlated with coping strategies such as positive reinterpretation and growth, humor, and planning. At the same time, they were positively correlated with coping strategies such as the focus on and venting of emotions, religious coping, and behavioral disengagement. No differences were found between the patients with MDD and BD regarding somatization scores. Compared to the general population, i.e., the healthy control group, patients with depression had significantly lower scores for positive reinterpretation and growth, humor, and planning, with higher scores for religious coping and behavior disengagement. Thus, we can assume that these coping strategies are more probably influenced by the depressive episode (state-dependent) rather than by personality (trait-dependent). In the case of focusing on and venting of emotions, this coping mechanism was more frequently used by subjects with BD compared to the control group, so it might also be state-dependent.

The SCL-90-R obsession–compulsion score correlated negatively with positive reinterpretation and growth, humor, and the focus on and venting of emotions. It correlated positively with behavioral disengagement. No differences were found between the patients with MDD and BD with respect to obsession–compulsion scores. Both the patients with MDD and BD showed lower scores for positive reinterpretation and growth, compared to the control group. Therefore, positive reinterpretation and growth are most likely state-dependent in the case of our patients. The patients with MDD also showed lower scores for humor and higher scores for behavioral disengagement. Therefore, the depressive state may influence the choice of coping strategy in the cases of humor and behavioral disengagement.

The SCL-90-R scores for interpersonal sensitivity were negatively correlated with coping strategies such as positive reinterpretation and growth, active coping, denial, and humor, and positively correlated with behavioral disengagement. In our study, the patients with MDD had significantly higher scores than those with BD for interpersonal sensitivity. Thus, patients presenting this symptom cluster were more frequently the ones with MDD. They also had lower scores for positive reinterpretation and growth, denial, and humor, as well as higher scores for behavioral disengagement, than the general population. Again, we might assume that in the context of our study, these coping strategies are more likely to be state-dependent.

High scores on the SCL-90-R for depression were negatively correlated with positive reinterpretation and growth and planning. Simultaneously, they were positively correlated with behavioral disengagement. The patients with MDD had significantly higher scores than those with BD on the SCL-90-R when it came to depression. They had lower scores for planning and positive reinterpretation and growth, along with higher scores for behavioral disengagement, compared to the control group. It is possible that the depressive state might predispose the individual to adopt behavioral disengagement (dysfunctional coping) and use planning (a type of active coping mechanism), while using positive reinterpretation and growth (a passive functional coping mechanism) less when dealing with stress (state-dependent strategies). A study conducted by Paans and collaborators on patients with euthymic bipolar found that subclinical depressive symptoms negatively influence the active coping strategies [42]. Likewise, another study on coping strategies by Mao and collaborators showed a strong correlation between passive coping strategies and depressive symptoms [43].

The patients scoring high for anxiety on the SCL-90-R had low scores for humor and positive interpretation and growth, along with high scores for religious coping and behavioral disengagement. The patients who scored high for phobic anxiety on the SCL-90-R scale rarely used coping strategies such as positive reinterpretation and growth, in favor of mental disengagement. In other words, patients with anxiety respond to stressful life events with religious coping and behavioral disengagement and, sometimes, mental disengagement, if their anxiety is phobic in nature. No differences were found between the patients with MDD and BD in terms of experiment anxiety and phobic anxiety. Both the patients with MDD and BD had lower scores for positive reinterpretation and growth than the general population. Patients with MDD had high scores for behavioral disengagement and religious coping. Again, we may assume that in the case of anxiety, the coping mechanisms are influenced by the episode. When it comes to phobic anxiety, mental disengagement as a coping strategy may be trait-dependent, as both the patients with MDD and BD had lower scores than the control group for mental disengagement.

The SCL-90-R scores for paranoid ideation were positively correlated with strategies like focusing on and venting of emotions. With no differences between the patients with MDD and BD for this item, in the case of the patients with BD, who have higher scores than the control group for focusing on and venting of emotions, the coping strategy is state-dependent. The hostility item on the SCL-90-R correlated negatively with the following coping strategies: positive reinterpretation and growth, humor, and planning. Both patients with BD and MDD had lower scores for positive reinterpretation and growth than the control group, while subjects with MDD also had lower scores for humor and planning. Therefore, with no differences between the patients with BD and MDD when it comes to the hostility item, positive reinterpretation and growth, humor, and planning seem state-dependent in the case of patients with MDD.

Psychoticism scores were negatively correlated with coping strategies such as positive reinterpretation and growth or active coping, and positively correlated with behavioral disengagement. No differences were found between the patients with MDD and BD regarding the scores for psychoticism. In the case of patients with MDD, who showed lower scores than the general population for positive reinterpretation and growth or active coping, and higher scores for behavioral disengagement, these coping strategies may be state-dependent.

According to Carver (2010), personality and coping both act independently and synergistically in regulating mental health [44]. A study performed by Nielsen and Knardahl, using the Brief Cope and HSCL, found that some coping strategies, such as religion, humor, and the use of emotional support, show a high stability in time (two-year time period), while others are less stable (i.e., behavioral disengagement and denial). In addition, the authors state that the level of distress prompts specific models of coping strategies [30]. In our study, the majority of the coping strategies seem to be state-dependent in the specific case of certain symptomatic clusters. Tsujii and collaborators found that remitted patients with depression used fewer task-oriented and more avoidance- (denial and behavioral disengagement) and emotion-oriented (religious) coping strategies [17]. As we found that the inpatients with MDD used predominantly religious and behavioral disengagement strategies for stress adjustment, this may imply that these strategies could be both state- and trait-dependent. The possibility that they may be both state- and trait-dependent should also be taken into consideration.

The number of episodes was positively correlated with the dysfunctional coping strategy of substance use. A study from 2020 by McHugh and collaborators supports the idea that substance use in stressful situations can be tied to the severity of mood symptoms, even though causality cannot be established [45]. In our study, the patients used passive functional and dysfunctional coping strategies more frequently than active functional ones.

As a limitation, our study was performed with a small number of patients, assessing their symptom clusters and coping strategies. The relatively small number of patients in our samples could make it difficult to generalize the results for the targeted groups. Moreover, we did not assess the coping strategies in the same remitted patients at discharge from the hospital or longitudinally in the ambulatory setting between two episodes. As the trait- or state-dependent paradigms for understanding coping strategies are still debated, further research is needed. One possibility would be to compare our results with those provided by the same patients after full remission of the acute episode. Also, more objective assessment methods would improve the reliability of the data.

It is noted here that self-reported psychological states without inputs from medical care providers, such as the SCL-90-R, carry inherent biases, including self-report bias, social desirability bias, recall bias, and emotional state bias, all of which can impact the accuracy and reliability of the results [46,47]. Other limitations of the SCL-90-R include the inability to replicate factor structure, linear vs. ordinal scale, limited stability of the nine-dimensional structure, and confounding variables, among others, in the available literature. [48,49]

Despite increasing interest in the role of coping strategies in mood disorders, most prior studies have either focused on unipolar depression or examined coping in general terms, without correlating it to specific symptom dimensions. Furthermore, limited research has compared patients with bipolar and unipolar disorders in terms of both adaptive and maladaptive coping mechanisms, using validated scales like the COPE and SCL-90-R. The strengths of the existing available studies lie in highlighting the importance of psychological coping; however, they lack differentiation between trait vs. state influences, and often do not explore associations with clinical symptom clusters. This study addresses the above-mentioned gaps by comparing coping strategies across patients with BD and MDD, analyzing their associations with psychiatric symptom dimensions. This may inform more individualized therapeutic strategies, including a larger, more demographically diverse sample and incorporating personality assessments or resilience markers, both of which could enrich the current model. Exploring the impact of targeted psychoeducational or CBT-based interventions on these coping strategies may also help in developing personalized treatment plans aimed at reducing relapse rates and improving overall functionality.

## 5. Conclusions

Different syndromes of psychological distress, as evaluated by using the SCL-90-R, are associated with one or several dysfunctional coping mechanisms. The ability to positively reinterpret stressful events and find a benefit from an undesirable situation was negatively correlated with all types of psychiatric suffering (somatization, depression, anxiety, interpersonal sensitivity, hostility, and psychoticism). Behavioral disengagement (passivity) was associated with depression, anxiety, somatization, interpersonal sensitivity, and psychoticism. The focus on and venting of emotions was linked to somatization and paranoid ideation. The ability to use humor to combat stress was negatively correlated with somatization, interpersonal sensitivity, and anxiety. An orientation towards religion in the presence of passive behavior was associated with anxiety and somatization. The patients who used drugs as a coping mechanism as their strategy to combat stress had a significantly higher number of relapses. In our cross-sectional study, the coping strategies appear to be state-dependent rather than trait-dependent, although for some of them, both possibilities may be valid. The scientific novelty of this research lies in its comprehensive analysis of the interplay between psychiatric symptom dimensions (via the SCL-90-R) and specific coping profiles (via the COPE inventory) in both MDD and BD populations. From a practical standpoint, identifying maladaptive coping strategies offers valuable implications for clinical practice since it supports the use of tailored psychoeducational and cognitive behavioral interventions to foster more adaptive responses to stress, potentially reducing relapse risk.

## Figures and Tables

**Table 1:** The socio-demographic and clinical data of the samples.

	MDD		BD	
Socio-demographic data	n = 30	%	n = 30	%
Gender				
Male	8	26.7	10	33.3
Female	22	73.7	20	66.7
Area of residence				
Urban	16	53.3	24	80
Rural	14	46.7	6	20
Clinical data	M	SD	M	SD
Age at disorder onset (years)				
16–30	5	16.7	12	40
31–45	7	23.3	11	36.7
46–60	15	50	6	20
61–75	2	6.7	1	3.3
>75	1	3.3	0	0
Age at study entry (years)	54.43	11.87	44.80	13.12
Age at first episode (years)	46.06	13.48	34.83	12
Number of episodes	4.9	4.56	7.13	11.84
Average length of hospitalization (days)	20.56	6.9	16.03	6.27

Source: “Eduard Pamfil” Clinic of Psychiatry, Timișoara, Romania. MDD = major depressive disorder; BD = bipolar disorder; n = number of patients, M = mean; SD = standard deviation.

**Table 2:** SCL-90: comparative scores (BD vs. MDD).

n = 30	Diagnostic	Average	Standard Deviation (SD)	*p* (Independent Sample *t*-Test)
SCL 1: Somatization	BD	1.32	0.86	0.06
	MDD	1.79	1.05	
SCL 2: Obsessive–compulsive	BD	1.35	0.76	0.08
	MDD	1.75	0.99	
SCL 3: Interpersonal sensitivity	BD	1.06	0.66	0.02 *
	MDD	1.54	0.87	
SCL 4: Depression	BD	1.39	0.87	0.001 **
	MDD	2.17	0.90	
SCL 5: Anxiety	BD	1.32	0.89	0.09
	MDD	1.74	1.00	
SCL 6: Hostility	BD	1.12	0.55	0.85
	MDD	1.15	0.88	
SCL 7: Phobic anxiety	BD	0.94	0.93	0.28
	MDD	1.21	1.00	
SCL 8: Paranoid ideation	BD	1.10	1.04	0.71
	MDD	1.20	1.05	
SCL 9: Psychoticism	BD	1.05	0.75	0.27
	MDD	1.30	0.97	

* *p* < 0.05, ** *p* < 0.01.

**Table 3:** COPE: coping strategies comparative scores (BD versus MDD).

n = 30	Diagnostic	Average	Standard Deviation	*p* (Independent Sample *t*-Test)
COPE1: Positive reinterpretation and growth	BD	11.30	2.98	0.02 *
	MDD	9.60	2.44	
COPE2: Mental disengagement	BD	8.70	2.00	0.24
	MDD	8.10	1.93	
COPE3: Focus on and venting of emotions	BD	11.36	3.21	0.31
	MDD	10.43	3.82	
COPE 4: Use of instrumental social support	BD	11.70	2.83	0.03 *
	MDD	10.10	2.73	
COPE 5: Active coping	BD	10.96	2.89	0.007 **
	MDD	9.20	1.82	
COPE 6: Denial	BD	7.60	3.28	0.003 **
	MDD	5.46	1.83	
COPE 7: Religious coping	BD	12.06	4.05	0.02 *
	MDD	14.23	2.51	
COPE 8: Humor	BD	9.46	4.08	0.003 **
	MDD	6.56	3.16	
COPE 9: Behavioral disengagement	BD	7.60	2.14	0.002 **
	MDD	9.53	2.47	
COPE 10: Restraint	BD	10.26	3.65	0.28
	MDD	9.33	2.98	
COPE 11: Use of emotional social support	BD	11.76	3.25	0.47
	MDD	11.20	2.75	
COPE 12: Substance use	BD	5.73	3.76	0.22
	MDD	4.73	2.30	
COPE 13: Acceptance	BD	11.43	2.22	0.71
	MDD	11.23	1.92	
COPE 14: Suppression of competing activities	BD	10.10	2.72	0.06
	MDD	9.00	1.48	
COPE 15: Planning	BD	11.70	3.26	0.09
	MDD	10.20	3.49	

* *p* < 0.05, ** *p* < 0.01.

**Table 4:** Correlation matrix COPE-SCL-90.

	COPE 1	COPE 2	COPE 3	COPE 4	COPE 5	COPE 6	COPE 7	COPE 8	COPE 9	COPE 10	COPE 11	COPE 12	COPE 13	COPE 14	COPE 15	SCL 1	SCL 2	SCL 3	SCL 4	SCL 5	SCL 6	SCL 7	SCL 8	SCL 9
COPE 1	1																							
COPE 2	0.24	1																						
COPE 3	0.05	0.03	1																					
COPE 4	0.44 **	0.23	−0.04	1																				
COPE 5	0.68 **	0.26 *	0.20	0.41 **	1																			
COPE 6	0.45 **	0.37 **	0.09	0.17	0.42 **	1																		
COPE 7	0.01	0.14	−0.01	0.18	−0.03	0.16	1																	
COPE 8	0.55 **	0.20	0.12	0.31 *	0.45 **	0.34 **	−0.07	1																
COPE 9	−0.37 **	0.05	0.04	−0.37 **	−0.27 *	−0.09	0.14	−0.33 **	1															
COPE 10	0.46 **	0.25 *	−0.07	0.15	0.28 *	0.31 *	0.07	0.30 **	−0.08	1														
COPE 11	0.37 **	0.34 **	0.13	0.65 **	0.25 *	0.07	0.05	0.21	−0.18	0.08	1													
COPE 12	−0.05	0.17	0.18	0.10	0.01	0.12	−0.12	0.07	−0.12	−0.12	−0.007	1												
COPE 13	0.20	0.06	0.24	0.06	0.40 **	0.12	0.19	0.09	0.09	0.28 *	0.21	−0.19	1											
COPE 14	0.51 **	0.12	0.19	0.14	0.52 **	0.40 **	0.23	0.24	−0.07	0.45 **	0.02	−0.08	0.45 **	1										
COPE 15	0.59 **	0.09	−0.03	0.48 **	0.42 **	0.14	−0.02	0.39 **	−0.41 **	0.60 **	0.24	−0.04	0.20	0.31 *	1									
SCL 1	−0.35 **	−0.12	0.28 *	−0.18	−0.14	−0.24	0.27 *	−0.27 *	0.37 **	−0.09	−0.20	−0.08	0.06	−0.02	−0.28 *	1								
SCL 2	−0.36 **	−0.20	0.34 **	−0.09	−0.14	−0.25	0.20	−0.27 *	0.39 **	−0.17	−0.14	−0.06	0.07	−0.004	−0.24	0.70 **	1							
SCL 3	−0.45 **	−0.10	0.16	−0.17	−0.26 *	−0.28 *	0.17	−0.26 *	0.39 **	−0.15	−0.15	−0.07	0.002	−0.08	−0.17	0.57 **	0.74 **	1						
SCL 4	−0.34 **	−0.10	0.14	−0.13	−0.22	−0.25	0.18	−0.21	0.49 **	−0.10	−0.09	−0.10	0.07	−0.07	−0.28 *	0.64 **	0.77 **	0.72 **	1					
SCL 5	−0.41 **	−0.11	0.18	−0.12	−0.20	−0.18	0.25 *	−0.7 *	0.48 **	−0.08	−0.15	−0.12	0.09	−0.05	−0.22	0.71 **	0.85 **	0.69 **	0.82 **	1				
SCL 6	−0.48 **	−0.14	0.23	−0.17	−0.20	−0.12	−0.003	−0.29 *	0.25	−0.18	−0.18	0.06	−0.03	−0.13	−0.25 *	0.43 **	0.56 **	0.53 **	0.42 **	0.55 **	1			
SCL 7	−0.35 **	−0.18	0.18	−0.14	−0.22	−0.17	0.02	−0.18	0.36 **	−0.16	−0.03	−0.22	0.05	0.03	−0.22	0.45 **	0.71 *	0.64 **	0.62 **	0.73 **	0.40 **	1		
SCL8	−0.22	−0.04	0.27 *	−0.03	−0.20	−0.04	0.17	−0.17	−0.03	−0.04	−0.04	−0.12	0.07	0.08	−0.02	0.42 **	0.55 **	0.59 **	0.46 **	0.52 **	0.49 **	0.48 **	1	
SCL 9	−0.39 **	0.06	0.06	−0.12	−0.27 *	−0.21	0.10	−0.20	0.37 **	−0.04	−0.18	−0.05	−0.04	−0.04	−0.12	0.55 **	0.66 **	0.68 **	0.70 **	0.78 **	0.48 **	0.64 **	0.61 **	1

* The correlation is significant at the 0.05 level (2-tailed). ** The correlation is significant at the 0.01 level (2-tailed).

## Data Availability

Data supporting the reported results are available in the database of the “Pius Brinzeu” County Clinic Emergency Hospital, where this study was performed.
